# The novel KLF4/PLAC8 signaling pathway regulates lung cancer growth

**DOI:** 10.1038/s41419-018-0580-3

**Published:** 2018-05-22

**Authors:** Yunlu Jia, Xiaogang Ying, Jichun Zhou, Yongxia Chen, Xiao Luo, Shudu Xie, Qin chuan Wang, Wenxian Hu, Linbo Wang

**Affiliations:** 1 Department of Surgical Oncology, Sir Run Run Shaw Hospital, Zhejiang University, Hangzhou, China; 2 Department of Radiology, Second Affiliated Hospital, Zhejiang University, Hangzhou, China

## Abstract

Accumulating evidence suggests that placenta-specific 8 (PLAC8) plays an important role in normal cellular process and human diseases, including multiple types of human tumors, and its role is highly relied upon in cellular and physiologic contexts. However, there are no reports on its expression profile and biological roles during lung cancer development. In the current study, both the clinical implications and biological effects of PLAC8 in lung cancer (LC) progression were investigated, and we identified and described the novel Krüppel-like factor 4 (KLF4)/PLAC8 regulatory pathway in cancer progression. Elevated PLAC8 levels were positively correlated with tumor size, histological grade, and tumor node metasis (TNM) stage, and LC patients with high PLAC8 expression suffered poor outcomes. In vitro and in vivo assays further revealed that endogenous PLAC8 promoted cell proliferation and tumor formation. We also found downregulated PLAC8 protein in several LC cell lines following the induction of KLF4, and immunohistochemistry analysis of LC tissues by microarray indicated a potential inverse correlation between PLAC8 and KLF4 expression. Luciferase reporter analysis and chromatin immunoprecipitation assays determined that KLF4 negatively regulated PLAC8 promoter activity via directly binding to the promoter region. Furthermore, the growth inhibition resulting from KLF4 overexpression was partially rescued by ectopic PLAC8 expression. Together, our data uncovered a previously unidentified role of PLAC8 as a central mediator in LC progression. PLAC8 was transcriptionally repressed by KLF4, and the novel KLF4/PLAC8 axis may act as a promising candidate target for LC diagnosis and therapy.

## Introduction

Lung cancer (LC) is a common and prevalent malignant cancer worldwide, and it continues to be a leading cause of cancer-related death^1,2^. Despite curative treatment, later recurrence and metastatic spread in non-curable stages are common and negatively affect LC patient outcomes. Therefore, further investigations to reveal the biochemical pathways and potential molecules responsible for cancer progression appear to be the main ways to search for new therapeutic targets and improve clinical outcomes.

Placenta-specific 8 (PLAC8, Onzin) was first identified during a genome-wide analysis of gene expression in the placenta, where its expression was restricted to the spongiotrophoblast layer of the mouse placenta and was therefore called PLAC8^3,4^. PLAC8 was proven to be involved in various cellular physical processes (such as the regulation of immunity, cell differentiation, and apoptosis)^5^, and the control of various human diseases, including infectious diseases, diabetes, and tumors^6–9^. For example, PLAC8 was critical to human prostate cancer and pancreatic cancer growth and metastasis according to previous studies^10,11^. In colon cancer, PLAC8-overexpressing cells exhibited increased phosphorylated extracellular signal-regulated kinase 2, which led to elevated cell motility and cancer invasion^12^. PLAC8 also acted as a novel biomarker in liver carcinoma^13^, and PLAC8 recovery could suppress PI3K/Akt/GSK3b/Wnt/β-catenin signaling to reduce cell proliferation^14^. Overexpression of PLAC8 was associated with the malignant progression and patients’ poor prognosis in clear-cell renal cell carcinoma^15^. All these intriguing findings elucidated a pivotal role of PLAC8 in cancer progression and development. However, the precise function and underlying mechanisms of PLAC8 in LC progression remain unclear.

Various functions of Krüppel-like factor 4 (KLF4) in normal development and carcinogenesis have been widely investigated^16^. As a zinc-finger transcription factor, KLF4 was initially found to be highly expressed in postmitotic, terminally differentiated epithelial cells of the skin and intestine^17,18^. As one of four factors that induce pluripotent stem cells, KLF4 modulated cell fate reprogramming and self-renewal of embryonic stem cells^19,20^. KLF4 plays a complex role in human cancers, acting as both a tumor suppressor and oncogene depending on the tissue type^21^. For example, ectopic expression of KLF4 resulted in the suppression of cell proliferation in LC, pancreatic cancer, gastric cancer, colorectal cancer, meningiomas, and cervical cancer^22–26^. An oncogenic role of KLF4 was identified in skin squamous cell carcinoma and melanoma^27–29^. A more recent study demonstrated that during tumor metastatic process, inactivation of KLF4 suppressed pre-metastatic niche formation and metastasis in perivascular cells^30^. KLF4 possessed a transactivation domain and a repression domain and could alter its positive or negative transcriptional function after binding DNA sequences with downstream promoter elements^31–33^. Our previous studies demonstrated that KLF4 was reduced in primary LC tissues and regulated cancer development and progression via the transcriptional downregulation of human telomerase (hTERT) and secreted protein acidic, rich in cysteine (SPARC)^34,35^.

In the current study, we aimed to determine the expression profile and the clinicopathological and prognostic implications of PLAC8 during LC development and reveal how endogenous PLAC8 expression regulates cancer cell growth. A novel KLF4/PLAC8 regulatory axis was also examined.

## Results

### Direct association of elevated PLAC8 expression with pathological features and poor overall survival in LC

**Fig. 1:**
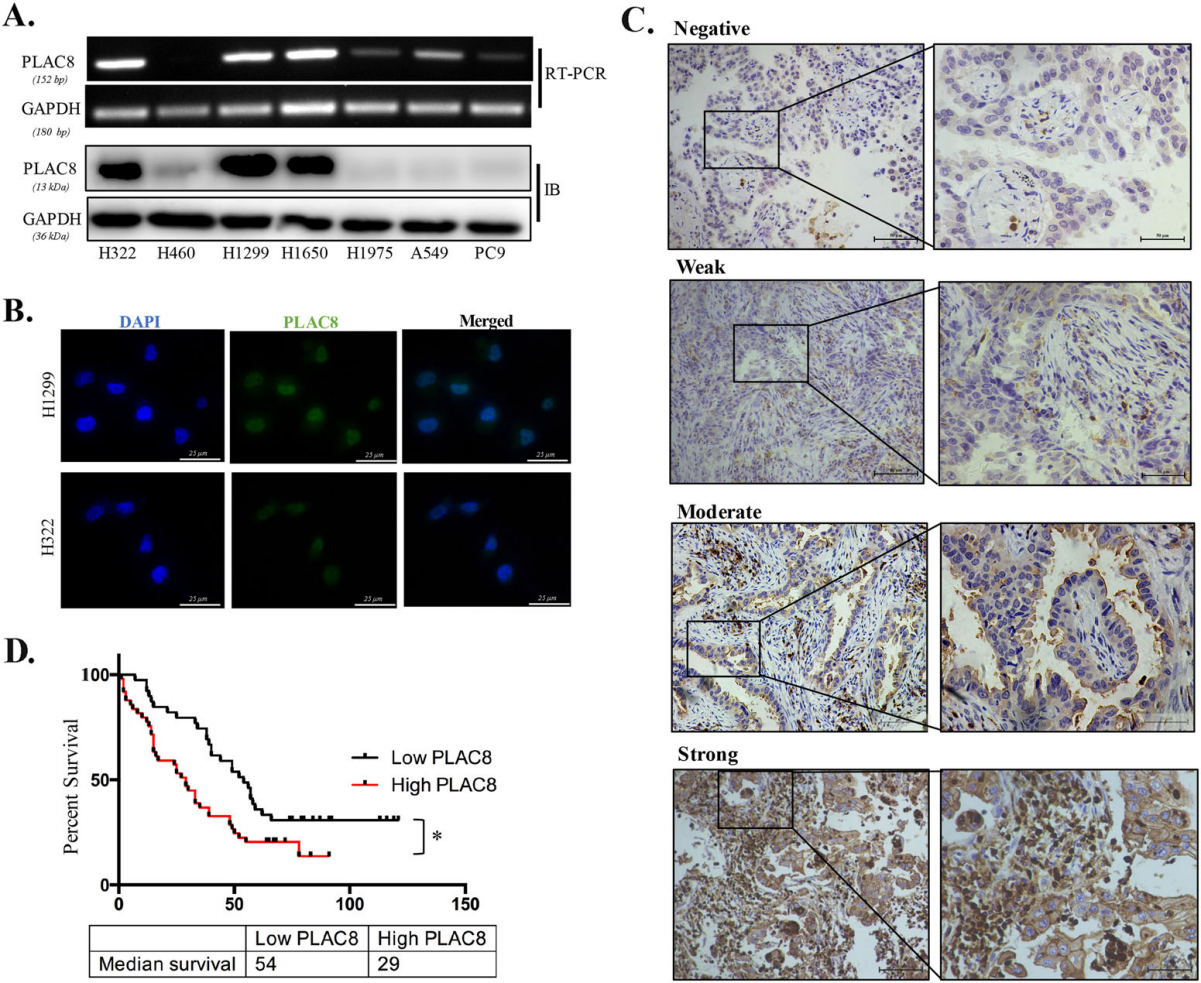
Expression of PLAC8 in lung cancer and its association with clinicopathological features in lung cancer TMAs. **a** PLAC8 mRNA (upper) and protein (lower) levels in seven LC cell lines were detected by Western blot and RT-PCR analysis. GAPDH was used as the loading control. **b** H1299 and H322 cells were stained for PLAC8 (green staining) and analyzed using fluorescence microscopy. Nuclei were stained with DAPI (blue staining). **c** Four representative images from the IHC analysis of PLAC8 expression (negative, weak, moderate, and strong) obtained from TMA LC samples. **d** The survival curve was compared using the Kaplan–Meier method according to the PLAC8 expression level. High PLAC8 was related to moderate and strong expression levels, while low PLAC8 was related to negative and weak expression levels. PLAC8 overexpression significantly predicted poor outcomes in LC patients (*P* = 0.0102). *P < 0.05

To gain initial insight into PLAC8 expression patterns in human tumors, we first screened the Oncomine database and conducted data mining in published cohorts (www.oncomine.org). Compared to that in normal tissues, PLAC8 mRNA was downregulated in most human cancer (Fig. S1A). In six published LC cohorts (Garber Lung, Landi Lung, Su Lung, Hou Lung, Selamat Lung, and Okayama Lung), we observed significantly decreased PLAC8 expression of LC, but show no difference in Wachi Lung, which may have been caused by the smaller sample size (*n* = 10) (Fig. S1B). However, the expression pattern of PLAC8 was interesting since excessive PLAC8 predicted malignant progression in LC patients (Fig. S1C). Furthermore, PLAC8 expression was analyzed in human LC tissue microarrays (TMAs) and LC cell lines. PLAC8 was highly expressed in H322, H1299, and H1650 cells, but expressed at relatively low levels in H460, H1975, A549, and PC9 cells (Fig. 1a). The localization of PLAC8 in LC cells (H1299 and H322) was confirmed using immunofluorescence staining, and PLAC8 was localized in both the nucleus and cytosol of LC cancer cells (Fig. 1b and Fig. S2). Additionally, correlations between PLAC8 expression and the clinical outcomes of 90 primary LC patients were examined. The baseline clinicopathological characteristics of TMAs are listed in Table 1. We observed PLAC8-positive staining in both the cytoplasms and nuclei of the LC specimens, and representive images of PLAC8 staining for four different cases were shown in Fig. 1c. Then, the PLAC8 expression levels were divided into two categories (low and high), and we investigated the relationships between the patients’ clinicopathological features and PLAC8 expression levels (Table 2). We did not find a remarkable difference between PLAC8 expression and the patients’ age, gender, LN invasion, or distant metastasis. However, the PLAC8 levels were closely correlated with the tumor size (*P* = 0.0400), TNM stage (*P* = 0.0460), and tumor differentiation (*P* = 0.0298). The overall survival rate of the PLAC8-high group was significantly worse than that of the PLAC8-low group (Fig. 1d). Consistently, univariate and multivariate survival analysis of our cohort revealed that increased PLAC8 expression was an independent prognostic factor for an unfavorable clinical outcome (Table 3). Collectively, these findings uncovered that PLAC8 might contribute to LC progression and may be a valuable biomarker for this disease.

**Table 1:** Baseline characteristics of lung cancer patients in the whole cohort

	Characteristic	Number (%)
Age (years)		
Median	61.5	
Range	30–84	
Gender		
Male	49	54.44
Female	41	45.56
Location of primary lesion, no. (%)		
Upper 1/3	42	46.67
Middle 1/3	10	11.11
Lower 1/3	31	34.44
Entire stomach	7	7.78
T stage		
T1/T2	67	74.44
T3/T4	23	25.56
N stage		
N0/N1	71	78.89
N2/N3	19	21.11
TNM stage		
I/II	46	51.11
III/ IV	44	48.89
Histologic grade		
I/II	53	58.89
III	37	41.11
Lymph node metastasis		
Negative	39	43.33
Positive	51	56.67
Distant metastasis		
Negative	88	97.78
Positive	2	0.22

**Table 2:** Correlation of PLAC8 expression to clinicopathological features in lung cancer

Parameters	PLAC8 (*n*)		
	−/+	++/+++	*P* value
Age (years)			0.3985
<60	14	21	
≥60	27	28	
Gender			0.1580
Male	19	30	
Female	22	19	
Histological grade			0.0298*
G1/G2	35	34	
G3	5	16	
Tumor size			0.0400*
T1/T2	34	33	
T3/T4	6	17	
LN metastasis			0.9762
N0	18	21	
N1	22	26	
Vessel invasion			
M0	41	47	0.3527
M1	0	1	
TNM			
1A–2B	26	20	0.0460*
3A–4	14	26

**Table 3:** Univariate and multivariate analysis of clinicopathological factors for OS and RFS

**Univariate analysis**		
Variables	OS	
	HR (95.0% CI)	*P* value
Age (years)	1.007 (0.979–1.035)	0.635
Gender	0.946 (0.557–1.605)	0.836
Tumor size	1.112 (0.632–1.958)	0.713
Vessel invasion	1.043 (1.131–8.277)	0.968
LN metastasis	2.251 (1.301–3.896)	0.004*
Grade	3.086 (1.036–9.187)	0.043*
PLAC8	2.360 (1.404–3.965)	0.001*
**Multivariate analysis**		
LN metastasis	2.321 (1.365–3.946)	0.002*
Grade	2.943 (1.035–8.366)	0.043*
PLAC8	2.317 (1.402–3.830)	0.001*

Note: Univariate analysis: log rank; multivariate Cox proportional hazards analysis

*OS* overall survival, *RFS* relapse-free survival, *HR* hazard ratio, *CI* confidence interval, *LN* lymph node

* Statistically significant (*P* < 0.05)

### PLAC8 silencing inhibited cell growth in LC

**Fig. 2:**
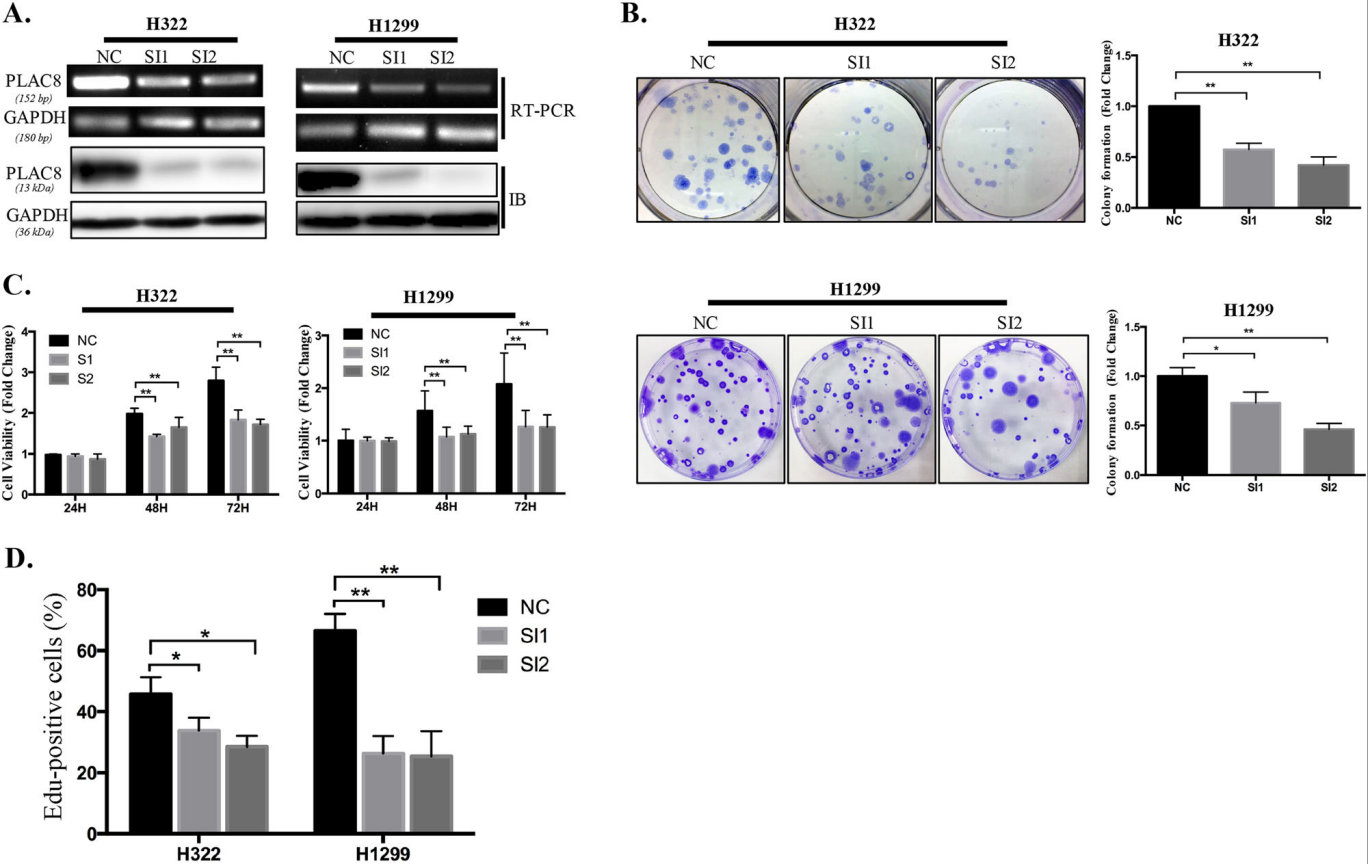
PLAC8 silencing inhibited H322 and H1299 cell growth. **a** The mRNA (upper) and protein (lower) expression of PLAC8 was downregulated in the PLAC8 knockdown cell lines H322 and H1299 compared with that in the negative controls. GAPDH served as the endogenous control. **b** Representative images of colony formation assays of the H322 and H1299 cell lines. PLAC8 silencing decreased the colony-forming efficiencies in both cell lines. **c** Knockdown of PLAC8 decreased the viabilities of the H322 and H1299 cell lines. **d** Quantification of the EdU incorporation rates in the PLAC8-silenced and control H322 and H1299 cell lines. Each bar represents the mean ± SD of three independent experiments. *P < 0.05 and **P < 0.01

To further evaluate the function of PLAC8 in LC development, we used two different small interfering RNAs (siRNAs) to silence PLAC8 in H322 and H1299 cell lines. The PLAC8 mRNA and protein levels were significantly reduced compared to the negative controls (Fig. 2a). The (3-(4,5-dimethylthiazol-2-yl)-2,5-diphenyltetrazolium bromide) tetrazolium (MTT) assay revealed that the knockdown of PLAC8 inhibited H322 and H1299 cell viability (Fig. 2c) and decreased cell proliferation and growth in a colony formation assay (Fig. 2b). A 5-ethynyl-2′-deoxyuridine (EdU) incorporation assay revealed that PLAC8 silencing in cells decreased their proliferation (Fig. 2d). These data demonstrated that PLAC8 silencing exerts an inhibitory effect on LC cell proliferation.

### Promotion of LC cell line growth by PLAC8

**Fig. 3:**
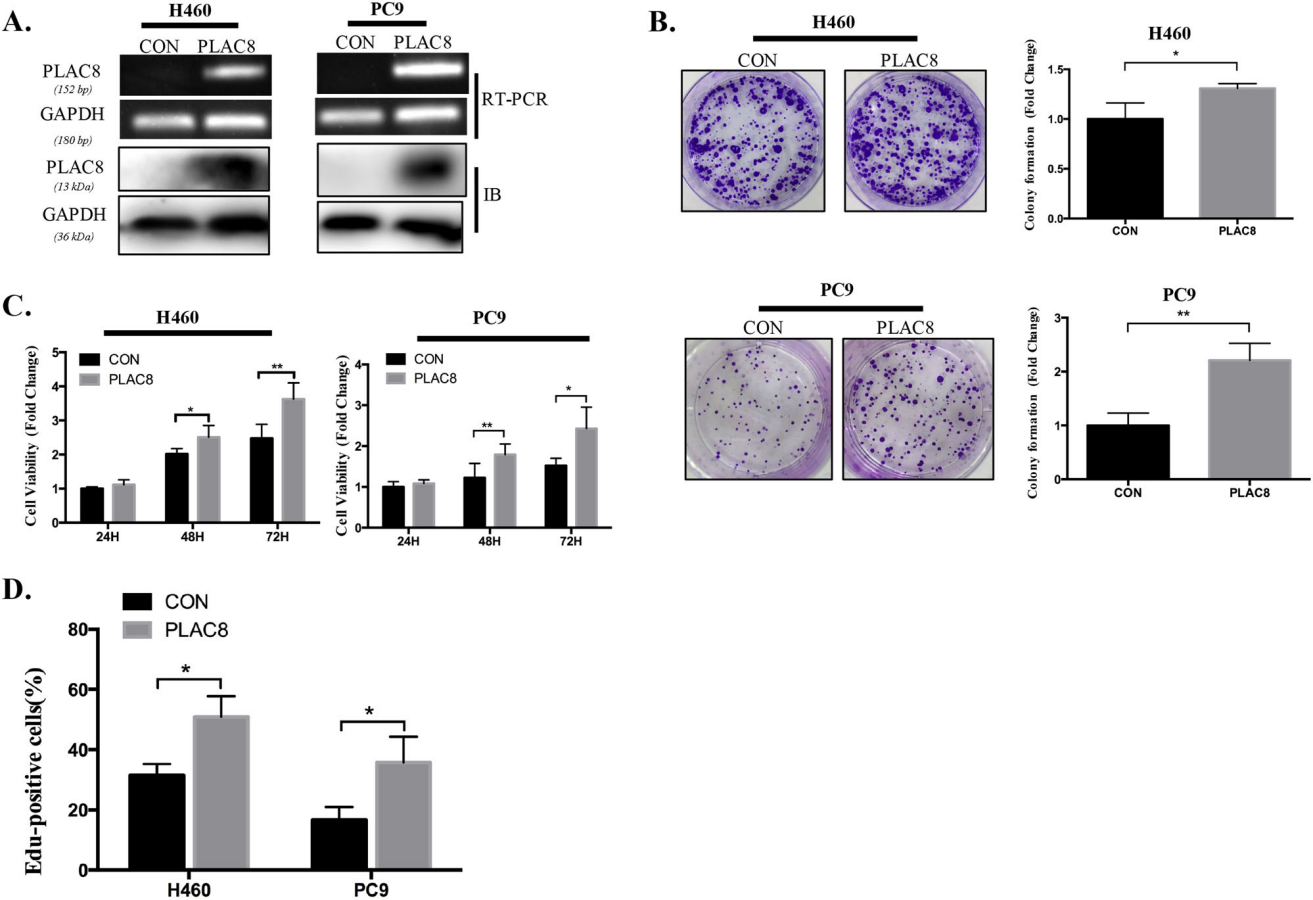
PLAC8 overexpression promoted lung cancer cell proliferation. **a** mRNA (upper) and protein (lower) expression of PLAC8 in the PLAC8-overexpressing cell lines H460 and PC9 and the negative controls. GAPDH served as the endogenous control. **b** Representative images of the colony-forming assays of the H460 and PC9 cell lines. **c** Overexpression of PLAC8 promoted the viability of H460 and PC9 cell lines. **d** Quantification of EdU incorporation (right) in PLAC8-overexpressing and control cells. Each bar represents the mean ± SD of three independent experiments. *P < 0.05 and **P < 0.01

We overexpressed the *PLAC8* gene using a lentiviral vector carrying the human *PLAC8* gene, and H460 or PC9 cells transfected with PLAC8-overexpressing and control vectors were used for the in vitro assays. The protein and mRNA expression levels of PLAC8 were remarkably elevated in H460 or PC9 cells after lenti-PLAC8 treatment (Fig. 3a). Quantitative analysis by the MTT assay revealed that the H460 and PC9 cell viabilities steadily increased following lenti-PLAC8 transfection (Fig. 3c), and colony-forming assays also indicated that PLAC8 overexpression promoted colony formation (Fig. 3b). An EdU incorporation assay showed that PLAC8 overexpression increased cell growth (Fig. 3d). These results suggested that PLAC8 overexpression significantly promotes the proliferative potentials of LC cells.

### PLAC8 overexpression effectively promoted lung tumor formation in vivo

**Fig. 4:**
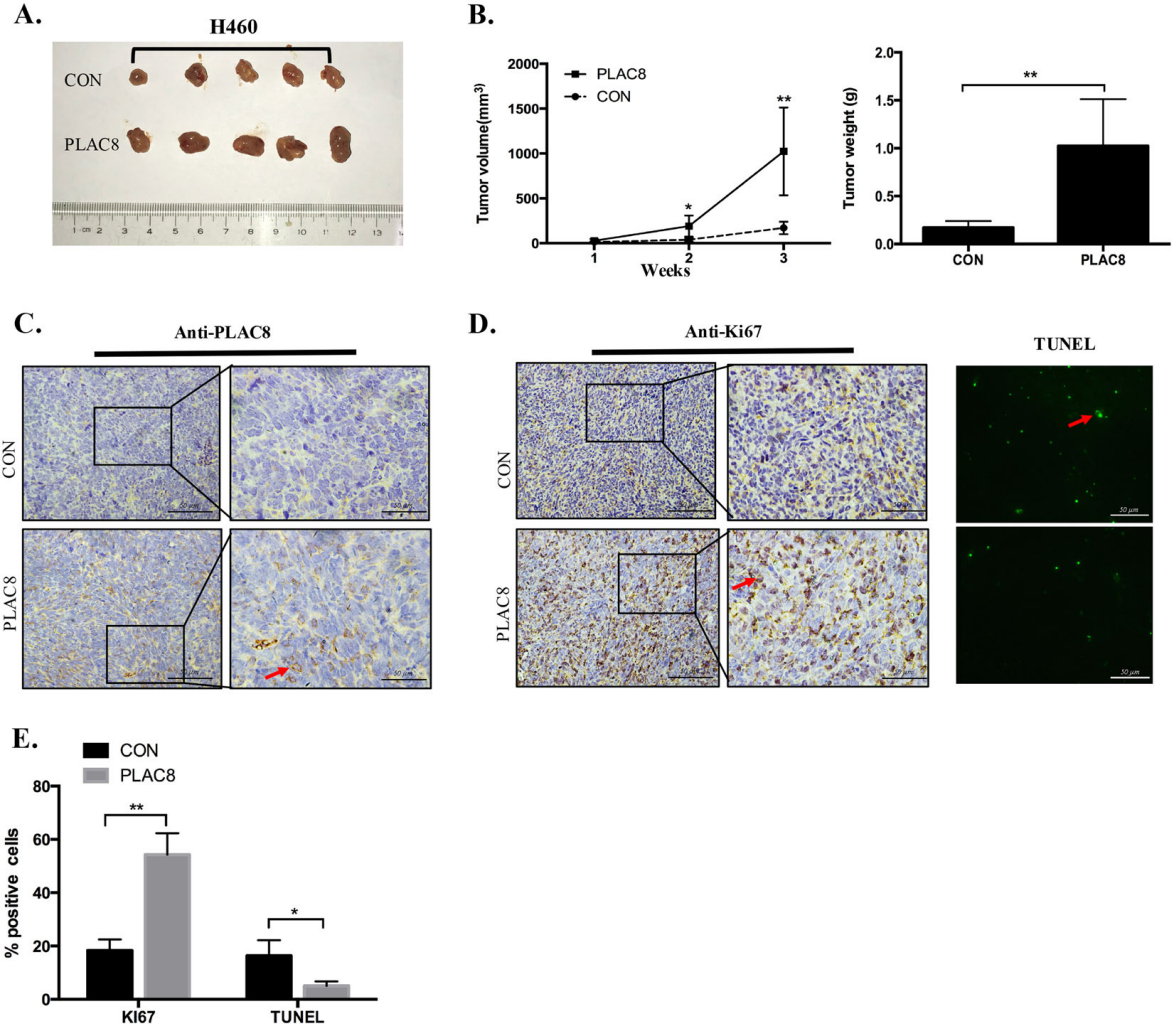
PLAC8 overexpression promoted the growth of lung cancer in vivo. **a** Representative tumor grafts are shown, which were grown in nude mice injected with H460/CON and H460/PLAC8 cells. **b** Tumor weight and tumor volume were compared between the PLAC8-overexpressing and control groups. The tumor volume was calculated using the following equation: *V* = (width^2^ × length)/2. **c** Representative images of PLAC8 staining are presented in each group of nude mice using immunohistochemistry analysis. **d** Representative images of Ki67 staining are presented in each group of nude mice using IHC analysis. TUNEL assays to analyze the immunofluorescence of apoptotic cells from nude mice in each group are also presented. **e** Quantification of the percentages of Ki67-positive and TUNEL-positive cells was calculated from three random fields. Each bar represents the mean ± SD of three independent experiments. *P < 0.05 and **P < 0.01

In animal models, we observed that tumors in the PLAC8-overexpressing groups grew significantly faster and larger than those in the control groups (Fig. 4a). There were significant differences in tumor volume and weight between the PLAC8-overexpressing groups compared with those in the control groups at 21 days post-implantation (Fig. 4b). The PLAC8 expression levels in the two groups are shown in Fig. 4c. Results from the immunohistochemical (IHC) staining of Ki67 and immunofluorescence from the terminal deoxynucleotidyl transferase dUTP nick-end labeling (TUNEL) assay showed increased tumor proliferation and decreased apoptosis in the PLAC8-overexpressing groups (Fig. 4c–e). These data supported that PLAC8 acts as an oncogene in LC progression in animal models.

### Inverse relationship between the expression levels of KLF4 and PLAC8 in LC

**Fig. 5:**
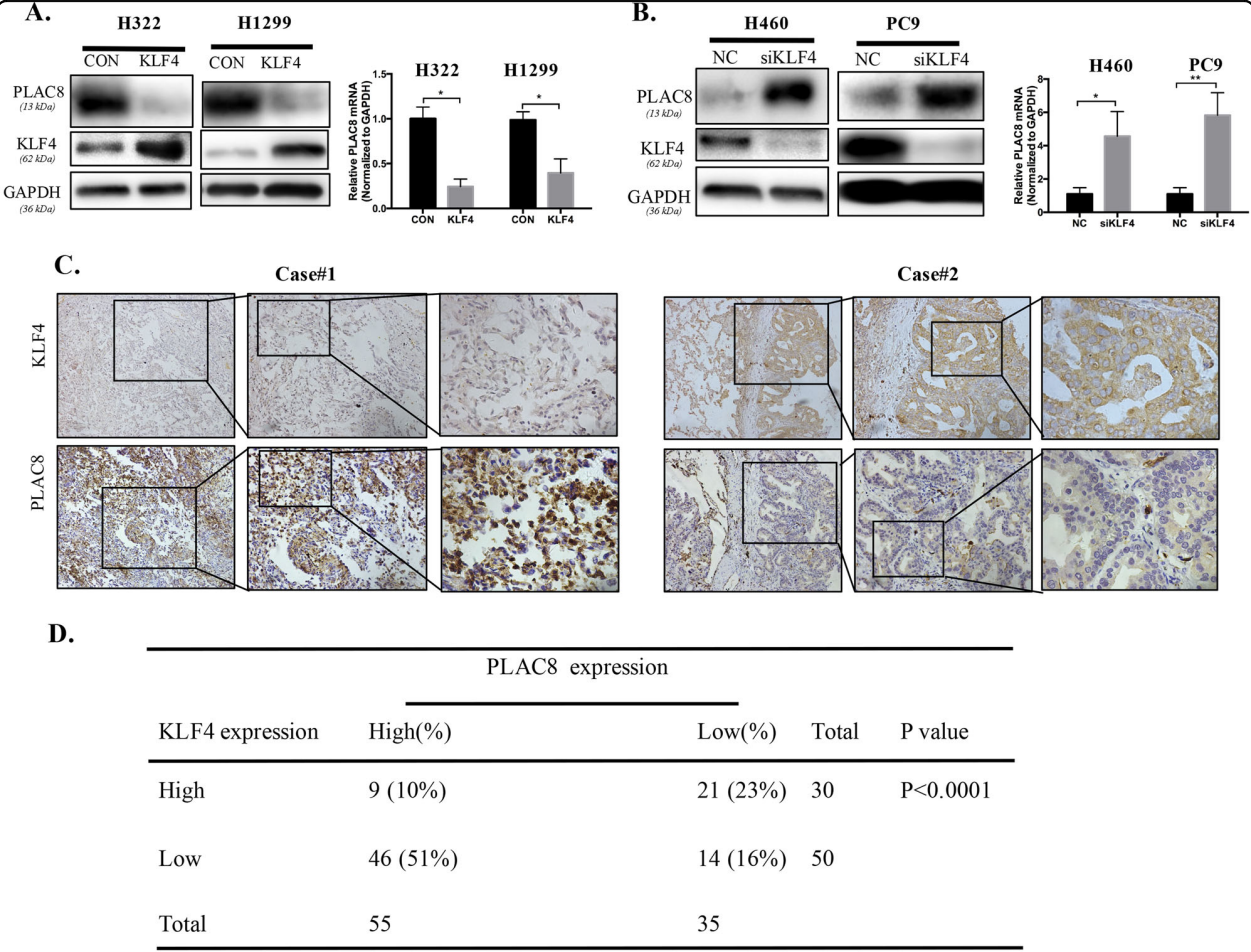
Co-expression of PLAC8 and KLF4 in lung cancer. **a**, **b** H1299 and H322 cells were transfected with KLF4 siRNA or control siRNA, and H460 and PC9 cells were transfected with Ad-KLF4 or the control for 48 h. Expression of PLAC8 and KLF4 was determined using Western blot and q-PCR analyses. Each bar represents the mean ± SD of three independent experiments. *P < 0.05 and **P < 0.01. **c** IHC analysis of LC TMAs for PLAC8 with a KLF4 antibody. Representative images of KLF4 and PLAC8 expression levels in LC samples are shown. **d** Correlation between KLF4 and PLAC8 expression levels in lung carcinoma tissues obtained from 90 patients.

As a transcription factor, KLF4 positively or negatively regulates its downstream targets after binding a DNA sequence and acts as a tissue-specific tumor suppressor or oncogene during tumorigenesis^36^. Here, we investigated the mechanisms underlying how PLAC8 promotes cancer progression and explored the effects of altered KLF4 expression on PLAC8 in LC cells. As shown in Fig. 5a, b, overexpression of KLF4 in H322 and H1299 cell lines decreased the expression of both PLAC8 protein and mRNA, whereas KLF4 knockdown markedly upregulated PLAC8 expression in H460 and PC9 cell lines. We further investigated KLF4 expression with the TMAs to analyze PLAC8 expression as described above. The results demonstrated that LC specimens with low KLF4 expression showed high PLAC8 expression levels (Fig. 5c). The correlation between the expression of KLF4 and PLAC8 in LC tissues was shown in Fig. 5d. Collectively, these data demonstrated that KLF4 negatively regulates PLAC8 expression, and the regulation most likely occurs at the transcriptional level.

### KLF4 directly bound the PLAC8 promoter and suppressed its expression

**Fig. 6:**
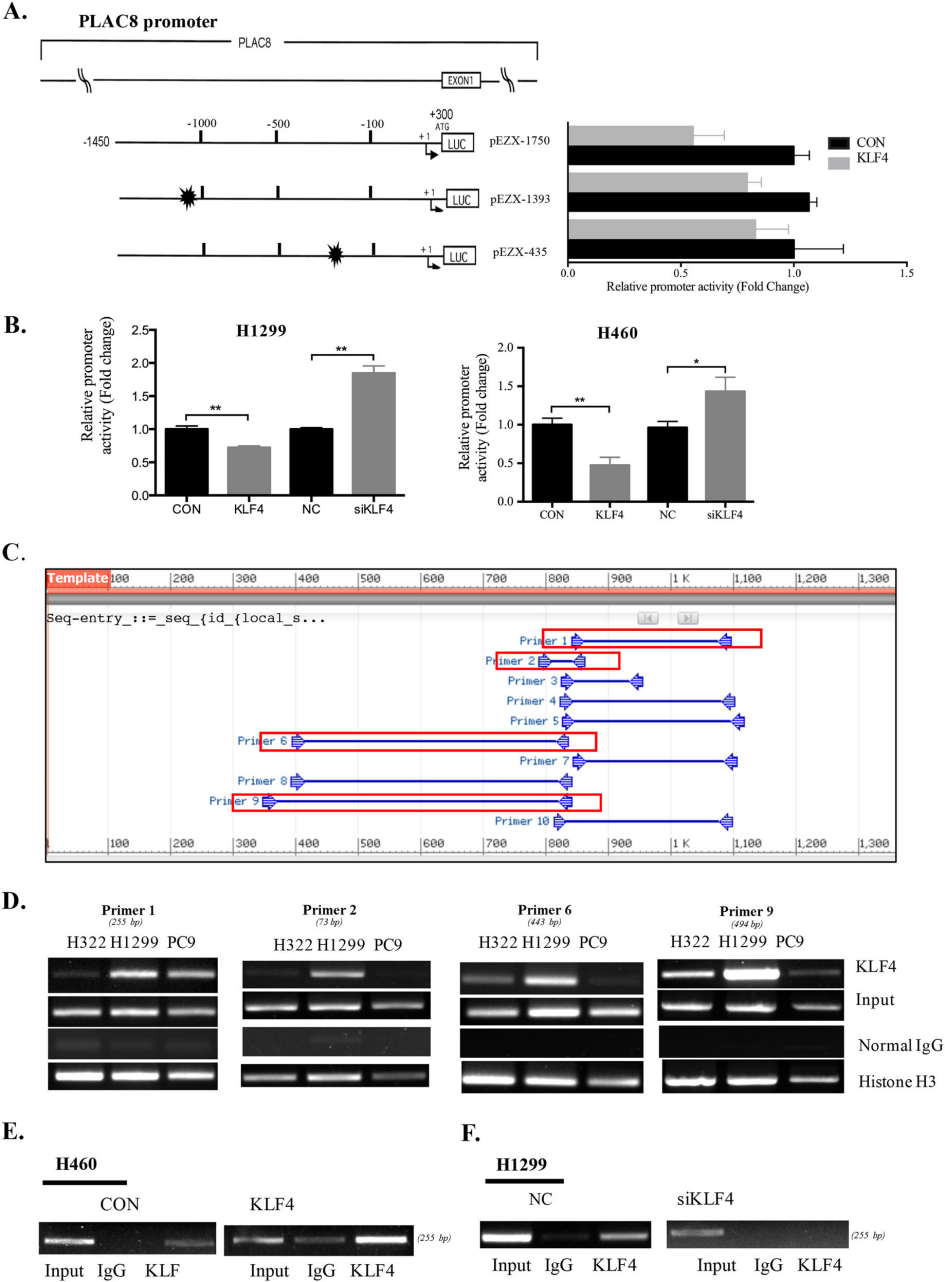
KLF4 directly bound to the promoter region of PLAC8. **a** Schematic representation of the structures of PLAC8 promoter luciferase constructs. Briefly, 1.7 kb DNA and 5′ truncated fragments of the PLAC8 promoter upstream of the initiating ATG were inserted into the luciferase (LUC) reporter vector pEZX-PG04.1 in the sense orientation. The arrow represents the transcription start site. The numbers represent the number of bases upstream (–) and downstream (+) of the transcription start site. Deletion mutation reporters for this plasmid (pEZX-1393 and pEZX-435) then were generated. Full-length PLAC8 promoter vector (pEZX-1750), deletion mutation reporters (pEZX-1393 and pEZX-435) with or without a KLF4 expression vector into H1299 cell lines. **b** H1299 and H460 cells were co-transfected with pEZX-1750 and a KLF4 expression vector or a control vector and KLF4 siRNA (si-KLF4) or control siRNA. The promoter activity in the cells was examined using a dual luciferase assay. **c** KLF4 putative binding sites in the PLAC8 promoter and primers used in the ChIP assay. **d** Chromatins were isolated from H322, H1299, and PC9 cells, and the binding of KLF4 to the PLAC8 promoter in these cells was determined using a specific anti-KLF4 antibody. **e**, **f** Results from a ChIP assay conducted using chromatins isolated from H460 cells transfected with Ad-KLF4 or a control plasmid and H1299 cells transfected with KLF4 siRNA or the control siRNA. Histone H3 was used as the positive control, and normal IgG was used as the negative control; 1% of the total cell lysates were subjected to PCR before immunoprecipitation (input control). Each bar represents the mean ± SD of three independent experiments. *P < 0.05 and **P < 0.01

To further explore how KLF4 negatively modulates PLAC8 expression in LC cell lines, a 1750-bp fragment DNA containing PLAC8 sequences from −1450 to 300 relative to the transcription initiation site was subcloned into the Pezx-PG04.1 vector. The resulting full-length reporter plasmid, which may contain multiple KLF4-binding sites, was designated pEZX-1750. Deletion mutation reporters for this plasmid (pEZX-1393 and pEZX-435) then were generated. We co-transfected a full-length PLAC8 promoter vector (pEZX-1750), deletion mutation reporters for this plasmid (pEZX-1393 and pEZX-435) with or without a KLF4 expression vector into H1299 cell lines (Fig. 6a). As shown in Fig. 6a, KLF4 inhibited the activity of all of the mutant reporters. We further co-transfected H1299 and H460 cell lines with pEZX-1750 and a KLF4 expression vector or KLF4 siRNA, and results in Fig. 6b indicated that ectopic KLF4 overexpression attenuated the PLAC8 promoter activity, whereas knocking down KLF4 expression activated the PLAC8 promoter activity. To determine the interactions between KLF4 and four potential KLF4-binding sites in the PLAC8 promoter region (Fig. 6c), a chromatin immunoprecipitation (ChIP) assay was performed in H322, H1299, and PC9 cell lines with an anti-KLF4 antibody (Fig. 6d). Moreover, KLF4 overexpression in H460 cells resulted in significantly increased chromatin fraction immunoprecipitation by the anti-KLF4 antibody, while H1299 cells transfected with KLF4 siRNA had the opposite effect (Fig. 6e, f). Taken together, these results suggested that KLF4 directly binds the PLAC8 promoter region and affects the transcriptional activity of PLAC8 in LC cell lines.

### Exogenous PLAC8 expression partially reverted KLF4-induced growth arrest

**Fig. 7:**
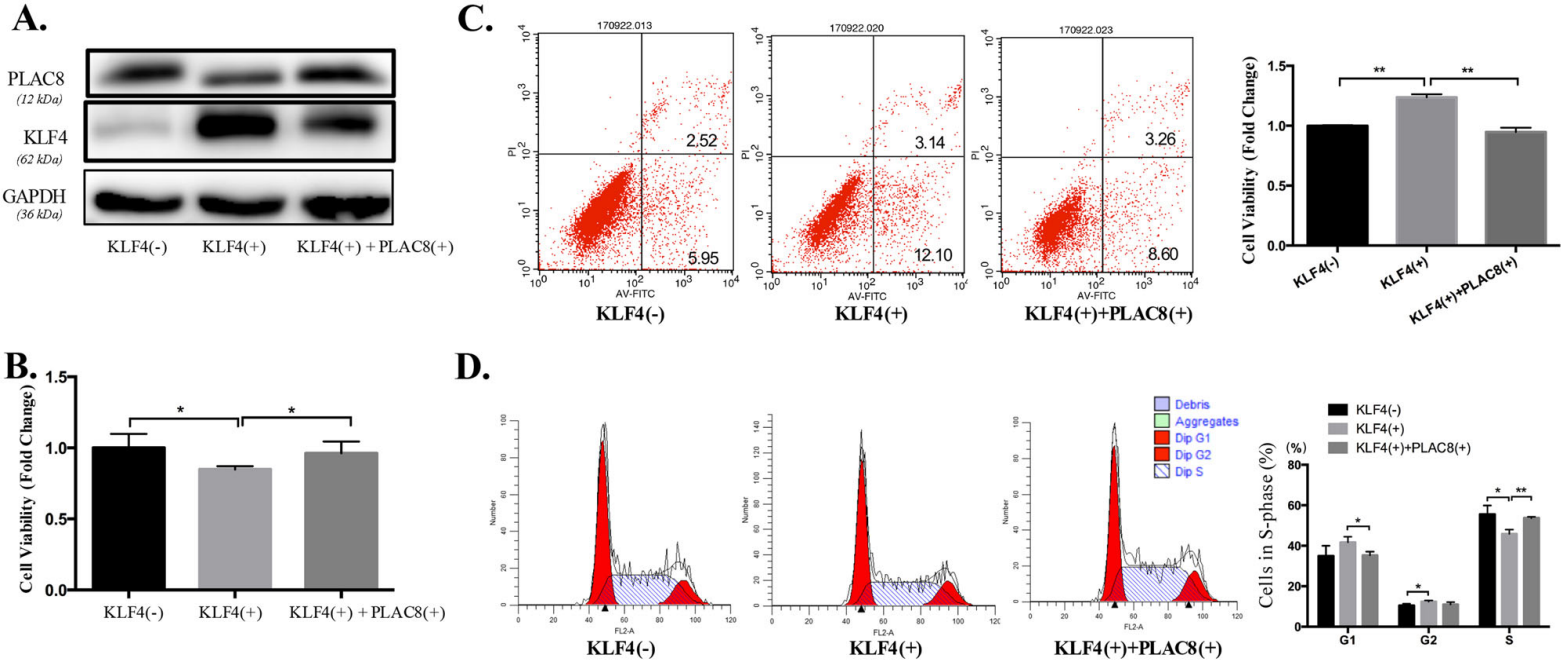
Exogenous expression of PLAC8 rescued the KLF4-induced inhibition of H1299 cell growth. **a** PLAC8 and KLF4 expression level in H1299 cell lines transfected with a KLF4-overexpressing vector or a PLAC8-overexpressing vector. GAPDH served as the endogenous control. **b** Exogenous PLAC8 expression partially reverted the growth arrest in H1299 cells, whereas KLF4 induced complete growth arrest. **c** Exogenous PLAC8 expression impeded KLF4-induced apoptosis. **d** KLF4-related cell arrest in the S phase was reverted by PLAC8 overexpression. Data are presented as the mean ± SD derived from three separate experiments. *P < 0.05 and **P < 0.01

We previously demonstrated that KLF4 inhibited cell proliferation and led to cell-cycle arrest at the G(1)–S checkpoint in LC^35,37^. KLF4 induced the downregulation of PLAC8 protein expression, and PLAC8 overexpression promoted tumorigenesis of LC cells. Next, we evaluated whether the KLF4-induced inhibition of cell growth was partially dependent on PLAC8 downregulation. First, we evaluated the PLAC8 and KLF4 expression levels in H1299 cells transfected with KLF4 or PLAC8 expression vector (Fig. 7b). As shown in Fig. 7a, PLAC8 overexpression in H1299 cells partially reverted the KLF4-induced slow growth of cells. Furthermore, the increase in cell apoptosis induced by KLF4 overexpression was significantly decreased in PLAC8-overexpressing cells compared with that in control cells (Fig. 7c). In a cell-cycle assay, PLAC8 overexpression increased the percentage of cells in the S phase and decreased the percentage of cells in the G1-phase in H1299 cells transfected with the KLF4-overexpressing plasmid (Fig. 7d). These data indicate that the effect of KLF4 overexpression on LC cell proliferation is partially dependent on PLAC8 downregulation, suggesting that PLAC8 is involved in the KLF4-mediated inhibition of cell growth.

## Discussion

In this study, we investigated the physiological function and clinical implications of PLAC8 in LC development and revealed a novel KLF4/PLAC8 signaling pathway. First, immunohistochemistry analysis of LC TMAs confirmed that PLAC8 expression had positive correlations with tumor size, tumor histological grade and clinical stage, and high PLAC8 expression predicted a shorter overall survival time in LC patients. Then, exogenous PLAC8 was validated to significantly promote cell proliferation and xenograft tumor formation both in vivo and in vitro. Additionally, luciferase and ChIP assays demonstrated that KLF4 directly bound and negatively regulated PLAC8 expression. As a downstream target gene of KLF4, PLAC8 partially rescued decreased cell growth rates by the inhibitory effects of KLF4.

Accumulating evidence suggests that PLAC8 has important functions in different cellular process and human diseases, and its functions differ by cell type. Both tumor suppressor and promoter roles of PLAC8 have been described in previous studies^38–41^. PLAC8 overexpression in rat fibroblasts suppressed apoptosis and promoted tumorigenic transformation^42^. However, the anti-apoptotic effects of PLAC8 were not found in primary acute myeloid leukemia cell lines, where PLAC8 contributed to inhibiting cell differentiation^43^. Moreover, PLAC8 overexpression induced cell apoptosis in both primary and transformed human lymphocytes^44^. Similarly, the effects of PLAC8 on cancer progression vary depending on the tissue type in other solid cancer development. Decreased PLAC8 expression was reportedly associated with hepatocellular carcinoma oncogenesis, and patients with a low level of PLAC8 suffered poor prognoses^45^. The oncogenic role of PLAC8 was reported in the early stages of pancreatic cancer, prostate cancer^10,11,46^, and colon cancer cells, exhibiting increased PLAC8 levels, which showed epithelial–mesenchymal transition features and resulted in tumor invasion^12^. A recent study demonstrated that PLAC8 expression was related to cell proliferation and prostate malignant transformation during chronic exposure to cadmium^47^. These results indicated that PLAC8 plays an essential role in cellular malignant transformation. PLAC8 has been identified as a co-operation response gene, and it could interact with other cellular components to execute three main functions: autophagy, cell-cycle arrest, and apoptosis^40,48^. Our own results added further evidence to the versatility of PLAC8 in cellular functions. We demonstrated that high PLAC8 expression was positively correlated with tumor progression and predicted poor outcomes in LC patients. Elevated PLAC8 expression promoted cell proliferation and tumor formation. These results strongly support the notion that PLAC8 functions as an oncogene in LC progression and represents a promising novel target gene for cancer treatment. We further investigated the potential co-operative transcription factor and underlying regulatory signaling pathway interacting with PLAC8 in LC progression.

KLF4 is a transcriptional factor that plays critical roles in cellular processes, including cell differentiation, development, proliferation, and apoptosis^49–51^. KLF4 also functions as a tumor suppressor or oncogene in various tumors^52^. For example, KLF4 has been shown to function as a tumor suppressor in LC^37^, lymphoma, cervical cancer^53^, neuroblastoma, pancreatic ductal gastric cancer, and hepatocellular carcinoma^54–57^. In breast cancer, the function of KLF4 is controversial, with reports showing both oncogenic and tumor-suppressive roles^58–61^. In addition, acting as a transcription activator or a repressor, KLF4 exerted its activation or inhibitory function after binding different target gene promoters^62,63^. Our previous studies revealed that KLF4 suppressed LC growth via directly binding to the promoter region of hTERT^35^. KLF4 may also regulate lung homeostasis and tumorigenesis via other mechanisms. Herein, we revealed a novel regulatory KLF4/PLAC8 pathway in LC progression. From immunohistochemical analysis of LC TMAs, there was a potential inverse correlation between PLAC8 and KLF4 expression levels. It was further demonstrated that KLF4 directly bound to the promoter region of PLAC8, and PLAC8 expression was transcriptionally suppressed by KLF4. Consistent with previous studies indicated, KLF4 inhibited the growth of many cancer cell lines, including LC cells^37,64^, and we further revealed that exogenous PLAC8 protects H1299 cells from KLF4-induced apoptosis and rescues the inhibitory effect of KLF4 on cell proliferation.

In this study, we obtained both clinical information and experimental evidence supporting PLAC8 as a key oncogene in LC progression. The expression of PLAC8 in LC was transcriptionally inhibited by KLF4, an important tumor-suppressive transcriptional factor during LC progression. This study not only identified a novel molecular target (PLAC8) during LC progression but also identified the aberrant KLF4/PLAC8 signaling pathway as a promising new molecular target for the design of novel therapeutic modalities to suppress LC progression.

## Conclusion

Collectively, we revealed that PLAC8 acts as an oncogene in the malignant progression of LC and a novel KLF4/PLAC8 signaling pathway that regulates tumor cell proliferation and apoptosis. KLF4 negatively regulates PLAC8 expression by directly binding to the promoter region of PLAC8, while PLAC8 partially rescues the suppressive function of KLF4 in LC proliferation.

## Materials and methods

### Cell culture

Cell lines were purchased from the Cell Bank of Chinese Academy of Sciences. The human LC cell lines (H322, H460, H1299, H1650, H1975, A549, PC9) were then stored in liquid nitrogen. These cells were cultured in RPMI-1640 medium (HyClone Thermo Fisher Scientific NYL1020) supplemented with 10% fetal bovine serum (Gibco) and 5% glutamine. The cells grew at 37 °C in a humid atmosphere containing 5% CO_2_.

### Plasmids, siRNAs, and transfection

Adenoviral KLF4 (Ad-KLF4, pcDNA3.1) was transfected into cells to overexpress KLF4. LacZ, a plasmid vector, was used as a nonspecific negative control. The previously described plasmids Ad-KLF4 and Ad-LacZ were gifts from Dr. Wenxian Hu (Sir Run Shaw Hospital, Zhejiang University)^37^. Lentiviral PLAC8- overexpressing and negative control vectors were gifts from Dr. Yongxia Chen. The full-length PLAC8 promoter vector and mutant PLAC8 promoter vectors were purchased and constructed from GeneCopoeia (product ID: HPRM35033). Retroviruses carrying siRNAs were used to knockdown KLF4 expression. Short interfering RNAs targeting PLAC8 and KLF4 (si-PLAC8 and si-KLF4) and a scrambled control siRNA were designed and synthesized commercially by RIBBIO (Guangzhou, China). Transfection of the plasmids and siRNAs into LC cell lines was conducted using the Lipofectamine 3000 (Invitrogen) transfection reagents following the manufacturer’s instructions. Cells were harvested for total RNA and protein extraction 48 h after transfection and processed for functional assays.

### RNA isolation and semiquantitative RT-PCR

Total RNA was extracted using Trizol Reagent (Invitrogen), and the RNA (1 μg) was reverse transcribed by the HiFiScript cDNA Synthesis Kit (CWBIO, CW2569M). Semiquantitative reverse transcription polymerase chain reaction (RT-PCR) was performed using 2× Taq MasterMix dye (CWBIO, CW0682S). Glyceraldehyde 3-phosphate dehydrogenase (GAPDH) was used as the reference gene. The following primers were used: PLAC8: 5′-GGAACAAGCGTCGCAATGAG-3′ (sense) and 5′- AAAGTACGCATGGCTCTCCTT-3′ (anti-sense); KLF4: 5ʹ-CGGACCACCTCGCCTTACA-3ʹ (sense) and 5ʹ-CTGGGCTCCTTCCCTCATCG-3ʹ (anti-sense); and GAPDH: 5ʹ-TGCACCACCAACTGCTTAG-3ʹ (sense) and 5ʹ-AGTAGAGGCAGGGATGATGTTC-3ʹ (anti-sense). The PCR products were loaded onto 1% agarose gels and visualized using ultraviolet light (Bio-Rad, Universal Hood II).

### Protein extraction and Western blot analysis

Protein extraction and Western blot analysis protocols were performed as described previously^35^. The following antibodies were used: PLAC8 antibody (1:1000, Cell Signaling Technology, #13885), KLF4 antibody (1:1000, Novous, IMG-6081A), Ki67 antibody (1:500, Sino Biological, 100130-T32-50), and GAPDH antibody (1:500, Santa Cruz, sc-47724). The signals were detected with an ECL Kit (Bio-Rad, Clarity™ Western ECL Substrate, 500 ml #1705061).

### Immunofluorescence staining

Cells at a density of 1 × 10^5^ were briefly plated in 6-well plates, and three glass coverslips were placed onto the wells. The plates were incubated for 24–48 h until 30–40% confluence was reached. The cells were then fixed with 4% paraformaldehyde for 10 min at room temperature and permeabilized with 0.1% Triton X-100. The slides were then washed three times with phosphate-buffered saline (PBS) and blocked with 5% bovine serum albumin in PBS for 30 min at room temperature. The sections were incubated with a primary antibody against PLAC8 overnight. After rinsing three times with PBST for 5 min, an Alexa 488-conjugated (green) goat anti-rabbit antibody was applied (1:200 dilution; Life Technologies) for 1 h at room temperature. Cell nuclei were counterstained with DAPI (4',6-diamidino-2-phenylindole) and images were acquired using a Nikon laser scanning confocal microscope (Nikon Instruments Inc., Melville, NY, USA).

### MTT assay

Cell viability was evaluated using the MTT assay (CellTiter 961 AQueous One Solution Cell Proliferation Assay, Promega). Cells at a density of 2 × 10^4^ were placed in a 96-well culture plate for 12 h, treated with MTT (5 mg/ml), and cultured at 37 °C for 4 h. Dimethyl sulfoxide was then added, and the mixture was oscillated for 5–10 min. The absorbance was then measured at 490 nm using a BioTek ELx800 absorbance microplate reader.

### Colony formation assays

Cells were seeded in 6-well plates at 500–1000/well and incubated for 10 to 14 days before staining with a crystal violet staining solution (Sigma-Aldrich). The colonies were then calculated and photographed.

### Flow cytometry for cell-cycle and cell apoptosis analysis

Cells were collected at 48 h after transfection with an siRNA or plasmid. Apoptosis was measured using the BD 556547 AnnexinV-FITC/PI Assay Kit following the manufacturer’s instruction. For cell-cycle analysis, cells were harvested and washed twice with PBS, and the Cell Cycle Staining Kit (MULTISCIENCES, CCS012) was used following the manufacturer’s instructions. Cell apoptosis and cell-cycle analyses were performed by flow cytometry (Accuri model C6).

### EdU incorporation assay

The proliferation of cancer cells was evaluated using the EdU keyFluor 488 In Vitro Imaging Kit (KGA331-500, KeyGEN Biotech, China). Cells were transfected with an siRNA or plasmid for 48 h, and an EdU reagent was then added at a final concentration of 20 mM and incubated at 37 °C for 2 h. All procedures were performed according to the manufacturer’s protocol. Five fields were randomly selected and observed under fluorescence microscopy. All images were processed using the ImageJ software, and the proportions of EdU-incorporated cells were counted.

### TUNEL apoptosis assay

The TUNEL apoptosis assay was performed following the manufacturer’s instructions (KeyGEN Biotech, KGA7052). The slides were treated with xylene for deparaffinization and rehydrated with ethanol; endogenous peroxidase activity was blocked with 3% H_2_O_2_ in methanol. A sufficient volume of proteinase K was added to completely cover the tissue sections, and the samples were incubated for 30 min in a humidified chamber at room temperature. After washing the slides three times with PBS for 5 min, 50 μl of TdT reaction buffer was added to each slide, and the slides were incubated for 60 min at 37 °C in the dark. The slides were washed with PBS three times for 5 min each, and 50 μl of streptavidin-FITC reaction buffer was added to each slide. The slides were then incubated for 30 min at 37 °C in the dark. Images were taken using a Nikon Ti inverted fluorescence microscope.

### Immunohistochemical staining

Tissue microarrays containing 90 pairs of human LC tumor specimens and normal lung tissue specimens were purchased from SOBC (HLug-Ade180Sur). Slides were stained with KLF4 and PLAC8 antibodies using the GTvisionIII Immunohistochemical Assay Kit (HRP/DAB, rabbit/mouse-general, two-step, GK500710, Gene Tech, Shanghai, China) according to the manufacturer’s protocol. Images were acquired by polarized light microscopy.

### Luciferase reporter assays

The indicated cells were seeded in 6-well plates and transfected with a PLAC8 reporter plasmid and KLF4-overexpressing vector, the negative control vector, KLF4 siRNA, or control siRNA. Forty-eight hours after transfection, the activities of Gaussia luciferase and secreted alkaline phosphatase in a dual-reporter system were measured using the Secrete-Pair Gaussia Luciferase Assay Kit (LF031, GeneCopoeia) following the manufacturer’s instructions; a 20/20 luminometer was used to acquire the activities of both.

### ChIP assay

ChIP assays were performed using reagents obtained from Cell Signaling Technology (SimpleChIP® Enzymatic Chromatin IP Kit (Magnetic Beads) #9003) as described previously^35^. Chromatin was used for immunoprecipitation with anti-KLF4, anti-histone 3, and a normal rabbit-IgG antibody. ChIP-enriched DNA was measured using real-time PCR, and the primer sets for the PLAC8 promoter were designed as follows: primer 1, 5′-GGTGAGGCGTGAATTCCTCT-3′ and 5′-TTGAGACGCCCATGAGACAC-3′; primer 2, 5′-GCTGAGAAAGGCCAAGCAAT-3′ and 5′-GGAGAGGAATTCACGCCTCA-3′; primer 6, 5′-CACTGGCAAGGGGATTGGTG-3′ and 5′-CTCAGGAGCAGTGTGAGAGTG-3′; and primer 9, 5′-CAAACCCTGTTCCTACACCAGT-3′ and 5′-CCCTTGCCAGTGTGTGAAGT-3′.

### Tumor xenograft assay

H460 cells were transfected with lentiviral PLAC8-overexpressing and negative control vectors. An equal number (2 × 10^6^) of cells were resuspended in 60 µl of PBS with 40 µl of growth factor reduced (GFR) basement membrane matrix (#356231 Corning) and then injected into 4-week-old nude mice (*n* = 5). The mice were observed three times a week, and the tumor volumes were measured twice per week. Tumor volume was calculated with the formula V = 0.5*ab*^2^ (*a*, longest tumor axis; *b*, shortest tumor axis). All mice were sacrificed 3 weeks after tumor cell injection. Tumor tissues were collected and processed for further analyses, including immunohistochemistry analysis of PLAC8 and Ki67 expression and TUNEL assay analysis of cellular apoptosis rates. Animal studies were reviewed and approved by the Ethics Committee for Animal Studies of Zhejiang University.

### Statistical analysis

The GraphPad Prism 6.0 software was used for statistical analysis. Data were independently collected from at least three experiments. *χ*^2^ tests were used for the evaluation of relationships between PLAC8 and KLF4 expression, and the PLAC8 expression levels and the clinicopathological features of LC TMAs. The survival curve was conducted using the Kaplan–Meier method with a log-rank test. Two-tailed unpaired Student’s *t* tests were used to compare mean data. The results are presented as the mean ± SD, and *p* < 0.05 was considered statistically significant.

## Electronic supplementary material

Figure S1

Figure S2

Figure S3

Supplementary figure legends
