# Supplementary figures and images for: The novel KLF4/PLAC8 signaling pathway regulates lung cancer growth
Source: Cell Death Dis. 2018 May 22;9(6):603. doi: 10.1038/s41419-018-0580-3 (PMC5964121; doi:10.1038/s41419-018-0580-3)

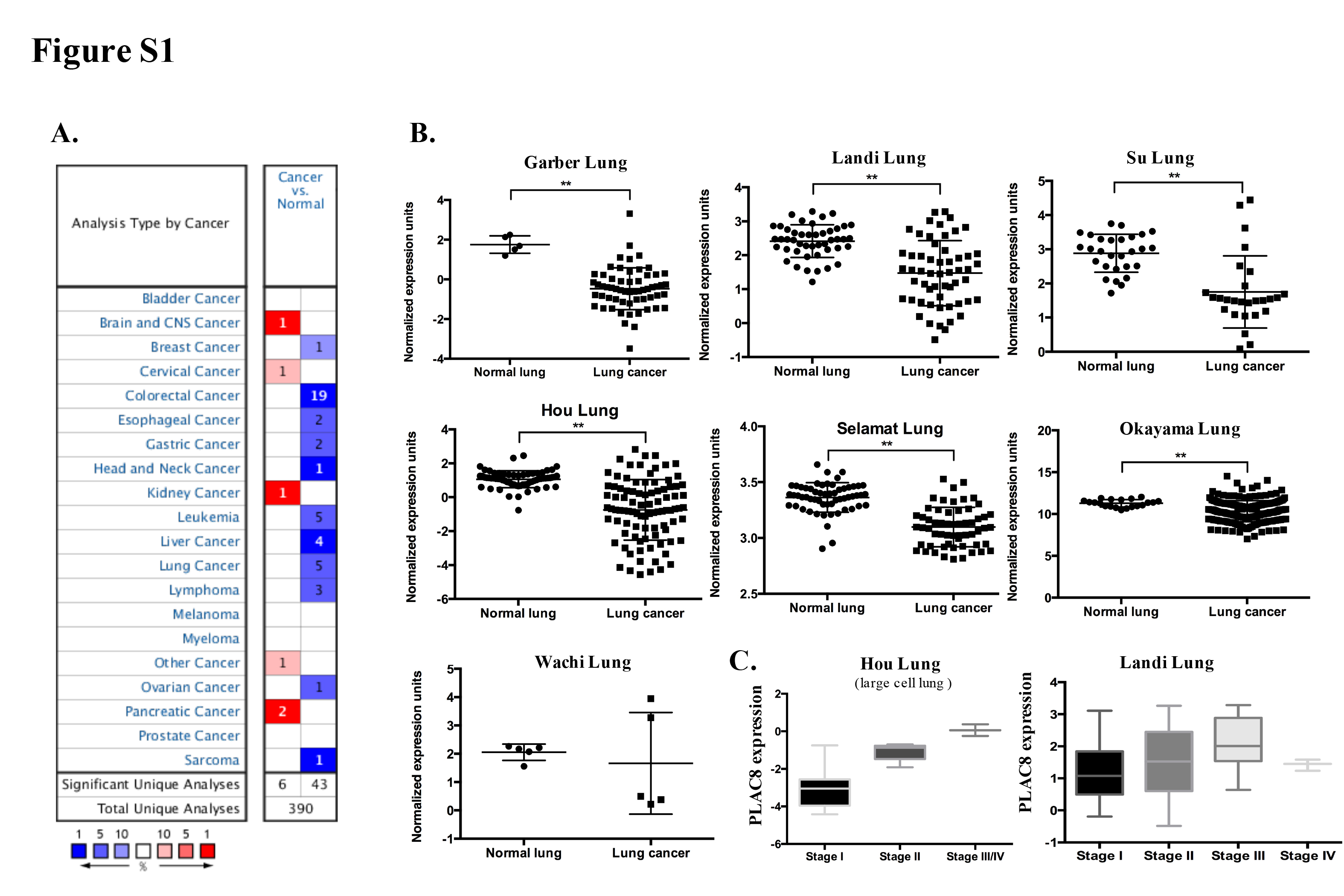

Supplement: Supplementary file 1 — Figure S1 [file 41419_2018_580_MOESM1_ESM.jpg]

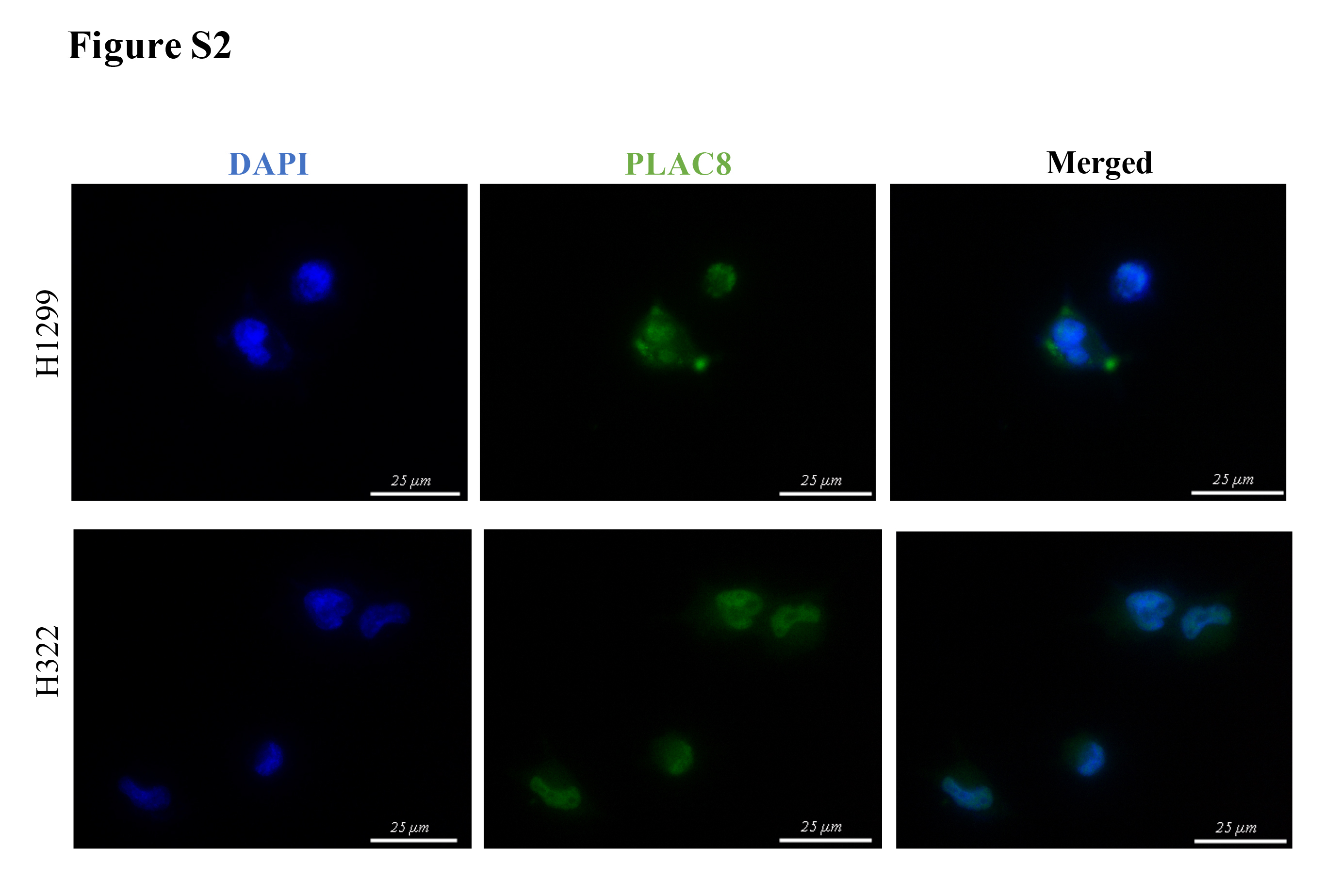

Supplement: Supplementary file 2 — Figure S2 [file 41419_2018_580_MOESM2_ESM.jpg]

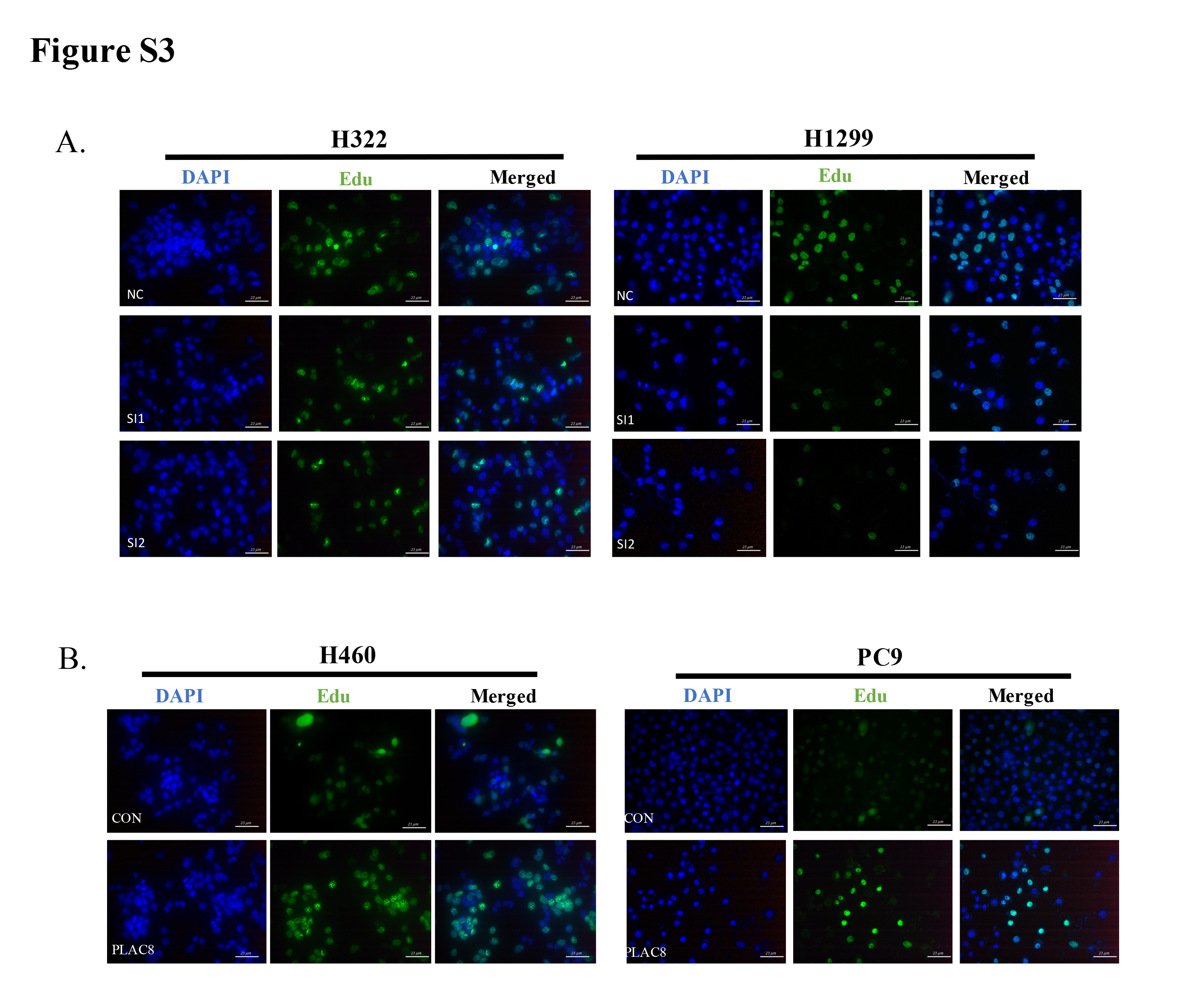

Supplement: Supplementary file 3 — Figure S3 [file 41419_2018_580_MOESM3_ESM.jpg]
