# Supplementary material for: The novel KLF4/PLAC8 signaling pathway regulates lung cancer growth
Source: Cell Death Dis. 2018 May 22;9(6):603. doi: 10.1038/s41419-018-0580-3 (PMC5964121; doi:10.1038/s41419-018-0580-3)
Supplement: Supplementary file 4 — Supplementary figure legends [file 41419_2018_580_MOESM4_ESM.docx]

Supplementary Figure S1

Alteration of PLAC8 expression in human cancers from Oncomine datasets. (A) mRNA expression of PLAC8 in human cancers and normal tissues (from Oncomine datasets). The red color indicates overexpression, while blue indicates underexpression. The number in the cell shows the amount of significant dependent studies. (B) Decreased expression of PLAC8 in tumor tissues compared with that in normal lung tissues in seven independent lung cancer studies (Oncomine, Garber Lung; Landi Lung; Su Lung; Hou Lung; Selamat Lung; Okayama Lung). Wachi Lung showed no significant difference in PLAC8 expression between lung cancer and normal lung tissues (P=0.64). (C) Positive correlation between PLAC8 expression levels and tumor stage in two single LC studies (Hou Lung Statistics (large cell lung) and Landi Lung). *P < 0.05, **P < 0.01.

Supplementary Figure S2

H1299 and H322 cells were stained for PLAC8 (green staining) analyzed using fluorescence microscopy. In all immunofluorescent panels, DAPI (blue) marks nuclei.

Supplementary Figure S3

(A). Representative micrographs of EdU incorporation rate in PLAC8-silenced and control H322 and H1299 cell lines (B) Representative micrographs of EdU incorporation in PLAC8-overexpression and control H460 and PC9 cell lines.
